# Rapid reduction versus abrupt quitting for smokers who want to stop soon: a randomised controlled non-inferiority trial

**DOI:** 10.1186/1745-6215-10-69

**Published:** 2009-08-14

**Authors:** Nicola Lindson, Paul Aveyard, Jackie T Ingram, Jennie Inglis, Jane Beach, Robert West, Susan Michie

**Affiliations:** 1 Primary Care Clinical Sciences, University of Birmingham, Edgbaston, Birmingham, B15 2TT, UK; 2 Public Health Nurse Specialist, South Birmingham Primary Care Trust, 6th Floor Triplex House, Eckersall Road, Kings Norton, Birmingham, B38 8SS, UK; 3 Health Behaviour Research Centre, Department of Epidemiology and Public Health, University College London, 2–16 Torrington Place, London WC1E 6BT, UK; 4 Centre for Outcomes Research and Effectiveness, Department of Psychology, University College London, 1–19 Torrington Place, London WC1E 7HB, UK

## Abstract

**Background:**

The standard way to stop smoking is to stop abruptly on a quit day with no prior reduction in consumption of cigarettes. Many smokers feel that reduction is natural and if reduction programmes were offered, many more might take up treatment. Few trials of reduction versus abrupt cessation have been completed. Most are small, do not use pharmacotherapy, and do not meet the standards necessary to obtain a marketing authorisation for a pharmacotherapy.

**Design/Methods:**

We will conduct a non-inferiority randomised trial of rapid reduction versus standard abrupt cessation among smokers who want to stop smoking. In the reduction arm, participants will be advised to reduce smoking consumption by half in the first week and to 25% of baseline in the second, leading up to a quit day at which participants will stop smoking completely. This will be assisted by nicotine patches and an acute form of nicotine replacement therapy. In the abrupt arm participants will use nicotine patches only, whilst smoking as normal, for two weeks prior to a quit day, at which they will also stop smoking completely. Smokers in either arm will have standard withdrawal orientated behavioural support programme with a combination of nicotine patches and acute nicotine replacement therapy post-cessation.

**Outcomes/Follow-up:**

The primary outcome of interest will be prolonged abstinence from smoking, with secondary trial outcomes of point prevalence, urges to smoke and withdrawal symptoms. Follow up will take place at 4 weeks, 8 weeks and 6 months post-quit day.

**Trial Registration:**

Current Controlled Trials ISRCTN22526020

## Background

Without help, most smokers who try to stop smoking relapse within one week and only 4% of quit attempts sustain abstinence for one year[1]. The best treatment results in about 22% one year prolonged abstinence[2], and the UK National Health Service (NHS) specialist stop smoking services achieve around 15% one year abstinence[3]. NHS support in primary care achieves around 7% one year prolonged abstinence[4]. Thus, while treatment improves substantially the number who achieve abstinence, whatever method of stopping is used, return to smoking is the norm for the majority and, even with treatment, the majority of relapsers resume smoking during active treatment. Thus we have a cadre of patients who have been through treatment services many times. Currently, the NHS gives the same treatment on repeated attempts to stop as on the first attempt. Patients often choose different pharmacotherapies, but in other respects, the treatment is the same every time. A common sense view is that offering repeated courses of identical treatment that failed previously might be less effective than trying different treatment. Rapid reduction might offer a new way to quit to those who have failed previously.

The standard assumption of all smoking cessation treatment is that cessation begins on a quit day and cutting down prior to quitting is not advised. This is because of the belief that with reduction each remaining cigarette will become more rewarding and harder to give up and in the meantime the smoker will suffer a loss of motivation before reaching the point where total abstinence is attained.

Smokers, however, feel that cutting down is an appropriate way to stop smoking. In the English Smoking Toolkit Study, 57% of current smokers reported they were cutting down, of whom 26% were using nicotine replacement therapy (NRT) to assist in this[5]. 40% of quit attempts were made by cutting down first. A review of epidemiological studies suggests that cutting down does not deter people from cessation, but is associated with an increase probability of trying to quit[6]. A survey of respondents to an advertisement of people interested in cessation found that 66% planned to stop by cutting down gradually, while 13% planned to stop abruptly. In a second survey of people who responded to an advertisement for those planning to reduce smoking, 57% planned to reduce then stop[7]. A survey of American daily smokers showed that 35% tried to stop gradually while 65% tried abruptly on their last attempt[8]. Those who chose gradual cessation were as motivated to stop and as confident of success as those who used abrupt cessation. They had the same level of dependence. A recently published random sample of American smokers showed that nearly half of smokers planning to quit would choose reduction over abrupt cessation and two thirds of these were interested in using medication to assist this[9]. In these smokers, there was little interest in reduction as an end in itself, only as a means to stop. Even among reducers not planning to stop soon, cessation was the goal of half. People who want to use gradual cessation to stop smoking soon have no NHS treatment programmes to assist with their attempt. Thus reduction as a means of stopping is likely to be popular, as well as providing another option for smokers who have tried and failed to stop previously.

### Literature Review

Observational studies have reported that those using gradual cessation were less likely to succeed than those using abrupt methods[10,11]. One reason may be, that those who chose gradual cessation as a means of quitting were less motivated to stop[12]. We therefore sought to find randomised trials. Based on Medline, Google Scholar, Psych Abstract, Society for Research on Nicotine and Tobacco abstract search, and a citation search plus contact with key authors, we identified the following trials as the only trials of abrupt and rapid reduction.

Marston & Mcfall randomised 65 students to one of four behavioural treatments, of which two were gradual reduction and abrupt cessation, but did not report smoking cessation rates[13]. In Gunther's trial, 55 smokers were randomised to reduction over five weeks and 55 to abrupt cessation after five weeks of behavioural support, followed by a further seven weeks of support[14]. The trial reports only relapse rates, but it is possible to calculate the relative risk (RR), 95% confidence intervals (CI) for unconfirmed undefined point prevalence abstinence at 12 months, which is 0.86 (0.44–1.68). Cinciripini randomised 128 people into four groups, scheduled reduction, non-scheduled reduction, scheduled smoking prior to quitting but no reduction, and normal smoking[15]. Baseline smoking rate was reduced by a third each week over three weeks. All groups received identical post-cessation cognitive behavioural therapy. The confirmed prolonged abstinence rates at 12 months were 44%, 18%, 32%, and 22% respectively. The Mantel-Haenszel (M-H) RR (95% CI) for reduction versus abrupt cessation is 1.14 (0.66–1.97). Flaxman randomised 64 participants to a 4 × 2 factorial trial[16]. Of relevance, two of the arms were gradual reduction and two abrupt cessation methods (crossed with aversive smoking, no aversive smoking). Post-cessation behavioural support was provided in each arm. The M-H RR (95%CI) for reduction versus abrupt cessation is 1.00 (0.47–2.13) for undefined unconfirmed point prevalence at six months. Cummings randomised 1895 smokers to a 2 × 2 factorial trial of daily versus unstructured self-help crossed with gradual versus abrupt quitting plan[17]. For unvalidated prolonged abstinence at six months, the M-H RR (95%CI) is 1.45 (0.87–2.42) for gradual cessation, based on exclusion of the 19% lost to follow up. The rates of loss were reported as 'fairly similar' in each arm. Cinciripini has recently completed a 3-arm study with participants randomised to cutting down with scheduled smoking and nicotine patch, nicotine patch pre-cessation treatment without reduction, or usual post-cessation patch only[18]. The results are unpublished, but preliminary data show improved cessation for the reduction arm at four weeks but no evidence of difference between the arms in abstinence at six months. The reduction arm had lower craving, fewer lapses, and less negative affect (personal communication). Hughes is also conducting a trial of reduction versus abrupt cessation with no results as yet.

A second group of studies have led to a new use of NRT for gradual cessation in a programme called Cut Down Then Stop (CDTS). Critically, the CDTS trials enrolled smokers who wanted to reduce their smoking but did not intend to stop it in the next one or six months. They were randomised to either NRT or placebo. The treatment programme gives NRT over 6–9 months for reduction and for a further three months post-cessation. The CDTS NRT programme led to improvements in six month prolonged abstinence over placebo (7% versus 3%), an RR (95%CI) of 2.06 (1.34–3.15)[19]. Six months prolonged abstinence is a standard accepted for evidence of effectiveness in smoking cessation[20]. Half of those who sustain abstinence for six months remain abstinent for their whole lives[21]. These cessation rates are lower than those achieved by the NHS smoking cessation services[22]. The key point is that these were rates achieved in smokers who said that they did not intend or want to stop imminently (and who were on average highly addicted). It is possible that the different intentions of smokers enrolled in CDTS trials and those that use the abrupt quitting method offered by the NHS explains the difference in prolonged abstinence rates (7% versus 18% six months prolonged abstinence). The CDTS studies demonstrate that reduction with NRT is more effective than without, but cannot show whether reduction or abrupt cessation is more effective.

The positive effect of pre-quit NRT has also been discovered in trials utilising the abrupt method of cessation, as outlined in the recent Cochrane review, which summarised trials that gave NRT patches for two weeks prior to quit day in addition to after cessation. The RR (95% CI) was 1.79 (1.17–2.72) compared with usual post-cessation use only[23]. In all these pre-cessation trials, NRT patch was used. Two studies used nicotine gum, with less effect[24,25]. Shiffman and Ferguson conducted a meta-analysis of four studies (three in the Cochrane review and one other) that all used pre-cessation patch treatments (1 of the studies used pre-quit nicotine patch (PQNP) for 4 weeks and the remainder used PQNP for 2 weeks). PQNP significantly increased the odds of abstinence both 6 weeks after quit date (Odds ratio (OR) = 1.96), and at 6 months (OR= 2.17)[26]. Participants were twice as likely to quit if they used PQNP, when compared to participants who started nicotine patch treatment on their quit day. Additionally post hoc observations found that in 3 of the 4 studies (relevant data were not available for the fourth study) participants treated with PQNP spontaneously reduced the amount of cigarettes they smoked, and those who reduced more of their own accord were more likely to quit. This may be because these participants were more responsive to treatment or could be further evidence that reduction can increase the chances of success in smoking cessation.

There are psychological principles that suggest that reduction might be more effective than abrupt cessation. One is shaping, obtaining a target behaviour (not smoking for the rest of one's life) by making successive approximations of the target behaviour (gradual cessation produces progressively longer periods of abstinence). The second is the cognitive psychology principle that completing a step toward a goal (reducing cigarettes per day) increases self-efficacy that increases the likelihood of completing the goal (abstinence). The third is classical and operant conditioning principles that decreasing the association of environmental cues (conditioned or discriminative stimuli) and a behaviour weakens the behaviour (smoking reduction typically uncouples cues from smoking). The fourth is the psychopathology principle that lowering drug intake reduces drug dependence increasing ability to abstain completely.

There is evidence that gradual reduction should be much more rapid than used in the CDTS trials. In one CDTS trial, Haustein (currently unpublished) participants were randomised in a 2 × 2 factorial design of NRT versus placebo and rapid versus slow reduction[27]. Despite enrolling people who said they did not intend to stop smoking in the next month, rapid reduction outperformed slow reduction at the end of trial (12 months from baseline). For confirmed 7-day point prevalence reduction, the M-H RR (95%CI) is 1.01 (0.55–1.84), confirmed prolonged abstinence it is 4.57 (1.00–20.93), and for confirmed prolonged reduction it is 2.69 (1.08–6.68). A pilot study randomised 31 smokers ready to stop to reduce over two weeks or three[28]. The quit rates were slightly higher in the two week group and qualitative data indicated that rapid reduction helped demarcate the boundary between reducing and quitting.

### Trial Objectives

#### Aim

To confirm or refute equivalence of two different behavioural instructions in stopping smoking, namely reduction or abrupt cessation.

#### Objectives

1) To measure smoking abstinence at 4 week, 8 week and 6 month follow-up in both the abrupt and reduction treatment arms.

2) To investigate possible mechanisms by which the behavioural advice to reduce prior to quit day achieves its effects.

## Method

All methods outlined below are in compliance with the Declaration of Helsinki as approved by the National Research Ethics Service (08/H0408/213)

### Why a non-inferiority trial?

Given reduction is so intuitive and appealing; it is likely to attract smokers who would have used abrupt quitting had reduction not been available. It is imperative to show that such smokers would not be worse off if they opted for a reduction programme and we propose to test this with a non-inferiority trial. Non-inferiority trials can show superiority but conventional superiority trials cannot show non-inferiority[29]. We propose an unblinded pragmatic trial large enough to show equivalence of the methods.

### Inclusion criteria

Participants must meet all of the following inclusion criteria to be eligible for enrolment into the trial:

1. males and females 18 years or older,

2. smokes at least 15 cigarettes or 12.5 grams of loose tobacco daily as roll your own cigarettes, or blows 15 parts per million or above on exhaled carbon monoxide (CO) reading.

3. willing to stop smoking completely in two weeks

4. evidence of a personally signed and dated informed consent document indicating that the subject has been informed of all pertinent aspects of the study and consents to participate and be randomised to either arm

5. be willing and able to comply with all study procedures.

### Exclusion criteria

Subjects presenting any of the following exclusion criteria will not be included in the trial:

1. currently using other NRT, bupropion, nortriptyline, mecamylamine, reserpine, or varenicline, or undergoing any treatment for tobacco dependence (e.g. acupuncture),

2. unstable angina pectoris, myocardial infarction, or cerebrovascular accident during the last 3 weeks,

3. severe cardiac arrhythmia

4. currently uncontrolled hyperthyroidism

5. active phaeocromocytoma

6. pregnancy, lactation or intended pregnancy

7. suspected alcohol or drug abuse

8. participation in other medicinal trials within the last three months and during study participation,

9. previously had severe skin reactions to nicotine patches or severe eczema or other skin diseases that make patch use hazardous or undesirable.

10. a severe acute or chronic medical or psychiatric condition or previously diagnosed clinically important renal or hepatic disease, that may increase the risk associated with study participation or may interfere with the interpretation of study results and, in the judgment of the investigator, would make the subject inappropriate for entry into this study.

### Withdrawal criteria

Trial Withdrawal: It is standard practice in smoking cessation trials to regard those who fail to attend for support and treatment as having relapsed, which is based on some evidence[20]. Therefore, failure to attend will not count as withdrawal from the trial and the only withdrawals will be those where a patient asks to be withdrawn. Such patients will not be replaced and, unless s/he refuses permission, data available up to that point will be used. Such withdrawals are expected in fewer than 5% of participants. This is standard procedure in smoking cessation studies.

Treatment withdrawal: One of our exclusion criteria is previous adverse reactions to NRT; so given that most smokers have used NRT recently, the established safety profile of NRT and the evidence from trials of combination NRT, we do not expect any serious adverse events due to the medication. Nevertheless, there will be a detailed work instruction for the trial that will detail the weekly assessment of side-effects, and the procedure for serious adverse events (SAE) and suspected unexpected serious adverse reactions (SUSARs). In the event of an SAE or SUSAR that is judged either possibly, probably, or definitely related to NRT, the prescription for NRT will be withdrawn and not re-instituted in that person.

### Participant Recruitment

We will recruit participants through South Birmingham, Solihull, Heart of Birmingham and Warwickshire and Worcestershire Primary Care Trusts in several ways. We will request that general practitioner's practices write to patients on their practice lists recorded as smokers and offer them treatment. We will also request that the stop smoking services write to people on their databases who have tried to stop and failed. The letters sent out will ask those patients who wish to take part in the trial to respond to the research team. In our experience, 5–10% will respond. Finally the trial treatment will also be offered to people booking for treatment with stop smoking services. Following telephone screening for preliminary eligibility, potential participants will be booked in for an assessment visit. Written informed consent will be obtained from participants at the first assessment session before they have been randomised to a treatment arm.

Participants in both treatment arms will be seen weekly for 2 weeks prior to quit day, on quit day, and weekly for 4 weeks post-quit. A further follow-up visit will take place 8 weeks post quit-day and a final follow-up phone call at 6 months. Therefore all participants will be enrolled in the trial for 6 months and 2 weeks.

It is not unusual for people to delay their quit day, once committed, for a variety of reasons, e.g. death of a close family member or friend. In this situation we will allow participants to delay their quit attempt for a maximum of 2 weeks. They will be advised to carry on with their pre quit NRT regime as prescribed and their actual quit date appointment will be recorded as their visit 3 appointment. Extra visits in between week 2 and week 3 will be recorded in the case record form and extra diaries and NRT will be issued to the participants to cover their delay period. Extra visit questionnaires will also be given to the participant for completion. Participants wishing to delay their quit day by >2 weeks will be classified as abandoning this quit attempt, NRT will be ceased and the participant will be advised to contact their local stop smoking service when they are ready to set a new quit date. They will be provided with the name and number of their local stop smoking service.

### Allocation to trial arms and treatments

#### Randomisation

Participants will be seen at an assessment session, similar to that used by stop smoking services, where participants will be randomised 1:1 to reduction or abrupt cessation in the assessment session. We will use Stata to accomplish stratified randomisation by therapist with blocking within each stratum to ensure balance. The blocks will be randomly ordered blocks of 2, 4, and 6. Each therapist will open sealed numbered envelopes in turn after consent and initial procedures to determine allocation to abrupt cessation or rapid reduction.

Participants in the reduction arm will be offered a choice of ways to reduce and asked to choose the method they feel is right for them. Those without strong preferences will be randomised to one of the three reduction methods. We will use Stata to accomplish stratified randomisation by therapist with blocking within each stratum to ensure balance. The blocks will be randomly ordered blocks of 3 and 6. Each therapist will open sealed numbered envelopes in turn after consent and initial procedures to determine allocation to abrupt cessation or rapid reduction.

It is quite likely that we will see couples, friends, or relatives who want to quit together and attend clinic as a group. In this situation they will be randomised together so they go into the same arm and given the same study number but made identifiable by adding an A or B to the end of their number.

#### Behavioural intervention in the abrupt cessation (control) arm

Participants randomised to abrupt cessation will have a brief discussion about smoking and about the nicotine patch treatment. Participants will be informed that the rationale of wearing the patch is to divorce the cigarette smoking behaviour from its reward (the delivery of nicotine). The relatively high constant level of nicotine is thought to blunt both the pharmacological cue to smoke and the reward from smoking when it occurs. The instruction will therefore encourage the smoker not to reduce consumption at all, even if they feel like smoking fewer cigarettes, because this will work against the stated rationale (not smoking will allow nicotine levels to fall to subnormal (for the participant) levels and create an urge to smoke that will be rewarded by smoking). Patches will be provided for use until the next visit and homework given to identify critical cigarettes, which is the basis for the pre-quit session discussion the next week, used in standard behavioural support. This will be followed by five weekly sessions on quit day and weekly thereafter, following the typical 7-session UK withdrawal orientated therapy programme[30]. Should a participant resume smoking during this treatment, participants will be allowed to renew their quit day following the new NHS standards. From quit day onwards participants in this arm will get combination NRT, meaning patch plus top-up acute product of their choice and be advised to use generous doses of NRT because dose is related to outcome[31] and combination treatment is more effective than patch alone[23]. Combination NRT is standard in our clinics and advised by the National Institute for Health and Clinical Excellence for dependent smokers[32]. Participants will be allowed to choose the type of acute NRT they prefer. Participants will be provided with a diary in weeks 1 and 2 to record the number of cigarettes smoked per day, and whether patches are being used, in week 3 this will be extended to include the amount of acute NRT product used per day.

#### Behavioural intervention in the rapid reduction (intervention) arm

The rapid reduction arm differs from the control arm only in the advice given in the pre-quit period two weeks before quit day. We will offer one of three reduction methods and the participants who do not have a strong preference for one or other method will be allocated at random to one of the three methods. The therapist will have a second set of sealed envelopes to accomplish this prepared as for the main randomisation. The two methods that focus on cigarettes per day will aim to reduce consumption to <50% of baseline at the end of the first pre-quit week and <25% by quit day.

The three methods are as follows:

a) Scheduled reduction (SR) method. In this method, a median inter-cigarette interval is calculated and then this altered so as to achieve the gradual reduction of cigarettes per day. For example, if a person is typically awake for 16 hours per day and smokes 16 cigarettes per day, then the median inter-cigarette interval is 1 hour. To achieve a 50% reduction, the inter-cigarette interval needs to increase to two hours. In this method, a person must smoke every two hours whether or not they want to do so. If they cannot smoke, that cigarette is missed and the next opportunity takes place two hours later. This method is potentially difficult in a country with smoke-free laws and for people with some types of jobs.

b) Hierarchical reduction. There are two variants: hierarchical reduction- difficult (HR-D) and hierarchical reduction- easy (HR-E). In this method, participants classify their usual cigarettes as either habitual cigarettes or particularly rewarding cigarettes. The HR-D method aims to get participants to reduce smoking by removing the difficult cigarettes first. The rationale is that getting rid of the hardest ones is the most difficult and if this can be accomplished well before total abstinence, this will enhance confidence and reduce the chance of a slip. HR-E is similar to HR-D except that participants seek to avoid smoking less rewarding or easier cigarettes to forgo. The rationale is that this gives participants early initial success and allows them confidence to tackle more difficult cigarettes later. If allocated to hierarchical reduction participants will be able to choose whether they will forgo easier of more difficult cigarettes first.

c) Smoke free periods (SFP). This method is different to all other methods in that it does not focus on cigarettes per day as the marker of reduction. In this method, participants map out their typical day, marking the smoking periods of the day. In a country with smoke free laws, smoking behaviour is concentrated into smoking breaks, except perhaps when at home. The SFP procedure will concentrate on reducing the number of smoking periods over the reduction time. In a smoking period, participants will be allowed to smoke as much as they want, but they will not be allowed to smoke outside the smoking period. The rationale is that smokers typically report few urges to smoke in places where smoking is forbidden, but find that not smoking when it is allowed more difficult. This method provides clear boundaries about when smoking is and is not allowed, unlike all other methods that depend on reducing cigarettes per day except perhaps scheduled reduction. It also focuses participants on what is being achieved- smoke free periods- and not on what is being forgone- cigarettes not smoked.

Each participant will complete a diary recording both the target for that day and a report on whether the participant met that target. In methods that focus on cigarettes per day, participants will be asked to put aside the next day's cigarettes into a separate pack to encourage adherence to the target. Participants will be instructed to replace cigarettes missed with a type of acute NRT (for example nicotine gum) and encouraged to use this sufficiently to avoid smoking more cigarettes than quota or smoking in a smoke-free period. Every evening, participants will record cigarettes smoked, acute NRT used (and the remainder in the packet), and note issues in a free text field. In a current trial, we find most participants complete the diary reliably.

### Trial Medication

The trial takes place within the context of NHS smoking cessation clinics, which provide behavioural support and medication to assist smoking cessation. Around 70% of smokers in these clinics use nicotine replacement therapy (NRT) as their medication to assist quitting and this is the only pharmacotherapy that will be available to participants in this trial. Current best practice is to use NRT in combination, which has been endorsed by the Medicines and Healthcare Regulatory Authority as good practice and recommended by the National Institute for Health and Clinical Excellence[32].

In this study, we propose using both nicotine patch and an acute form of NRT in combination (See Table 1). We will use Niquitin, Nicotinell, Wockhardt, and Nicorette NRT products, including 24 hour patches, which are licensed for 16 hour use also (there is no evidence that the effectiveness of 16 hour patches or 24 hour patches is different[33]). These patches provide about 1 milligram (mg) per hour of nicotine. Participants will be advised to wear the patch 24 hours per day, but will be advised to wear it only during day time should they experience sleep disturbance or vivid dreams. Although the medications provide the same amount of nicotine delivery with similar pharmacokinetics, the license dosing advice differs. Niquitin CQ recommend the 21 mg patch for smokers who smoke 10 or more cigarettes per day, while Nicotinell recommend the 30 cm^2 ^(= 21 mg) patch for smokers of 20 or more cigarettes per day. The evidence is that most smokers who smoke 10 or more cigarettes per day get less nicotine from their patch than they did from their cigarettes[34,35]. We have therefore chosen a cut-off of 15 cigarettes per day. In addition, all participants will be offered additional intra-nasal or oral nicotine replacement (gum, microtabs, lozenges, or inhalator) with the choice of delivery system left to personal preference. The dose of these products used will vary, but participants will be advised to take at least 1 mg of absorbed nicotine for each cigarette forgone in the reduction phase because each cigarette delivers about 1.2 mg on average, though this is highly variable[36]. A 2 mg oral product, for example gum, delivers about 1 mg available systemically, and a 10 mg inhalator cartridge yields about 3 mg of nicotine that is systemically available. In the cessation phase, participants in both arms will be given identical dosing instructions and advised to use at least 6 mg of absorbed nicotine daily, which is the minimum dose associated with improved outcomes[33].

**Table 1:** Daily medication regimes

	**Rapid reduction**	**Abrupt cessation**
**Pre-quit period -2 weeks to quit day**	21 mg/24 hour patch, 1 mg of absorbed nicotine per cigarette forgone as a minimum from acute NRT.	21 mg/24 hour patch, No acute NRT.

**Quit day onward**	21 mg/24 hour patch, Minimum of 6 mg of absorbed nicotine from acute NRT. As much as needed to feel comfortable.	21 mg/24 hour patch, Minimum of 6 mg of absorbed nicotine from acute NRT. As much as needed to feel comfortable.

### Rationale of the pre-quit NRT in the abrupt and rapid reduction arms

By utilising pre-quit NRT in both arms we will ensure that any effect is caused by the difference in reduction rather than differences in nicotine intake. Smoking and using a patch gives higher concentrations of nicotine than just smoking, whereas smoking and using an acute form of NRT does not[37]. It is thought that high levels of nicotine dissociate the cigarette from its reward and this is responsible for the effectiveness of nicotine pre-treatment, which is not apparent when acute forms of NRT only are used. Consequently, we have chosen a nicotine patch for both arms. However, gradual reduction could undermine the nicotine pre-treatment effect so we aim to keep nicotine levels high using a patch during reduction and replacing cigarettes with acute NRT.

### Duration of medication use and discontinuation

The license for NRT allows continued use for up to 9 months, but patch use will be phased out using the step down doses between 2 and 3 months after quit day. For participants who are still lapsing but showing determination to stop smoking, the patch will be phased out more slowly, providing no signs of overdose are evident. The dose reduction regimes vary in the summary of product characteristics and in any case the dose reduction is individual, based upon confidence in reducing the patch dose and occurrence of urges to smoke. Oral NRT is commonly continued for several months in abstinent smokers [38-41] and there are reasons to assume this is beneficial, and again we will apply clinical judgement in deciding on length of treatment of oral/intranasal NRT. The advice on patch duration and oral NRT discontinuation will be the same in both arms. At week +8 participants will be given a month supply of NRT and advised to contact either their general practitioner or local stop smoking service should they need further support and/or prescriptions beyond week +12.

### Stopping rules/modification of medication regime

• Participants who have problems with insomnia or difficulties with vivid dreams will use the patch for 16 hours daily, not 24 hours

• Participants who have skin reactions to the patch that are not controlled by switching preparations, emollient and hydrocortisone cream will switch to acute NRT only.

• Participants who become pregnant may have their dose adjusted in line with the National Institute for Health and Clinical Excellence guidance and in accord with the wishes of the participant.

• Participants who show symptoms of overdose will have the dose reduced.

• Participants who fail or give up on their quit attempt will cease using NRT.

### Evidence of safety of smoking whilst using NRT

The safety of using transdermal NRT while smoking was investigated in a review by Fagerstrom & Hughes[37], which is summarised in Table 2. In addition to this review, we have recently systematically reviewed the Cut Down to Stop (CDTS) studies mentioned in the literature review[19]. As part of this, we performed a meta-analysis of adverse events in people smoking and using NRT versus those smoking and using placebo NRT. Overall, 1384 predominantly middle-aged smokers were treated with NRT for 6 to 18 months, while 1383 were treated with placebo. Four deaths occurred in those randomised to NRT and four in those randomised to placebo; Odds ratio (OR) 1.00 95%CI 0.25–4.02. Serious adverse events occurred in fewer than 8% of participants in both arms; OR 1.09 95%CI 0.79–1.50. In no cases were these were judged likely to have been due to treatment. Discontinuation of treatment due to adverse events was rare with 1.7% and 1.3% in the NRT and placebo groups; OR 1.27 95%CI 0.64–2.51. Nausea was selected as an index symptom to indicate possible nicotine overdose. It was slightly and significantly more common in the NRT group with 8.6% versus 5.3% on placebo experiencing nausea; OR 1.69 95%CI 1.21–2.36.

**Table 2:** Summary of Fagerstrom & Hughes review of the safety of smoking and concomitant NRT use[37]

**Study reviewed**	**NRT treatment given**	**Blood nicotine concentrations**	**Safety conclusions**
Foulds et al, 1992	16+ cigarettes per day (cpd) & 15 milligram (mg) transdermal patch (TP) over 16 hrs	Baseline: 37 nanograms per millilitre (ng/ml), Placebo: 36 ng/ml, 15 mg TP: 44 ng/ml.	Participants experienced almost no subjective toxic effects whilst wearing the patch

Pickworth, Bunker & Henningfield, 1994	13+ cpd & 22 mg, 44 mg TP over 24 hrs	Baseline: 30 ng/ml, Placebo: 19 ng/ml, 22 mg TP: 39 ng/ml, 44 mg TP: 63 ng/ml.	No adverse subjective experiences were reported.

Mahmarian et al, 1997	8+ cpd & 14 mg TP over 24 hrs	Baseline 16 ng/ml, 14 mg TP: 24 ng/ml, 21 mg TP: 30 ng/ml.	Only adverse effects noted were nausea & vomiting in 2 patients.

Zevin, Jacob & Benowitz, 1998	Smoking ad libitum & 21 mg, 42 mg, 63 mg patch over 8 hours	Placebo: 20 ng/ml, 63 mg TP: 60 ng/ml.	No additional haemodynamic effects of TP on heart rate, blood pressure, noradrenaline, white blood cell count, fibrinogen, haematocrit, cortisol, or lipids. No adverse reactions.

Carpenter et al, 2000	11+ cpd & TP, gum or inhaler	Lower than 22 mg TP: 54% increase, Higher than 22 mg TP: 190% increase.	Number of cpd reduced by 43% and CO by 31%.

### Lifestyle advice

There is no special dietary or life-style advice that is imposed by using NRT and the associated regimes for using it proposed in the protocol. However, participants using oral NRT will be advised to avoid acidic drinks 15 minutes prior to using oral NRT.

### Concomitant Medication

All medications will be permitted for use concurrently, except those that are proven to help smoking cessation (bupropion, nortriptyline, mecamylamine, reserpine, varenicline), or medications that are unlicensed and for which no interaction data with NRT are available. No rescue therapies will be permitted in treatment, in accordance with the National Institute for Health and Clinical Excellence guidance on smoking cessation pharmacotherapy[32]. The NRT itself is aimed at the relief of symptoms of nicotine withdrawal. Should adverse skin reactions occur with the use of the patch, advice will be given on the use of over the counter emollients and 1% hydrocortisone cream, as is standard. Data on all concomitant medication will be recorded.

### Trial Outcomes

#### Primary trial outcome

• Abstinence at four weeks, measured according to the Russell standard[20,42]. The Russell standard allows a two week grace period from quit day for slips[43].

### Secondary trial outcomes

• Point prevalence at each follow up and prolonged abstinence at 8 weeks and 6 months. Half of those sustaining abstinence for six months sustain it for life, the goal of treatment[44,45].

• Urges to smoke and nicotine withdrawal symptoms will be measured after cessation. Urges to smoke are an important proxy of return to smoking.

### Other trial outcomes (non-efficacy)

• Exhaled carbon monoxide using a CO monitor is a measure of smoke inhalation. We will compare smoke exposure before quit day in both arms of the study.

• Cotinine levels, to show whether nicotine intake rises from normal when smoking in both arms, as we expect, and whether the rise in cotinine relates to the success of treatment. We propose measuring cotinine at baseline, week -1, quit day, and at week +1 on all participants to examine whether reduction leads to higher self-medication with nicotine. It could be that reduction treatment gets people used to using high doses of NRT and therefore some of the effect of treatment could be explained by post-quitting NRT dose. These data will also provide valuable evidence on nicotine consumption while smoking and using NRT.

• Participant rating of the reward from their cigarette while smoking using the modified Cigarette Evaluation Questionnaire (mCEQ)[46,47]. The mCEQ measures satisfaction, taste, mood, cognitive, and sensory sensations to smoking particular cigarettes. Twice each week prior to quitting (to keep participant burden reasonable), we will ask participants to rate satisfaction from smoking from the first cigarette of the day and one other key cigarette. We routinely ask participants to rate cigarette satisfaction in one specimen day so as to anticipate danger periods for lapsing after cessation. Typical rewarding cigarettes are after dinner. Additionally, participants will be asked to rate a cigarette smoked in a negative affect situation. It is possible that the mechanism of benefit may be the reward from smoking, and this study will allow investigation of this. We will also rate satisfaction from smoking after cessation, should slips occur.

• Confidence in quitting is a predictor of abstinence and might be modified by reduction and will be used in mediation analysis. This will be measured by a single question used in other trials that has been shown to predict success. How high would you rate your chances of giving up smoking for good at this attempt? (Circle one response)

Extremely high

Very high

Quite high

Not very high

Low

Very low

• Smoking stereotypy is a measure of the degree to which smoking is prompted by cues to smoke[48]. Perhaps reduction might work by disrupting stimulus control and this scale could measure this. Two questions from this scale will be used to measure smoking stereotypy because the other questions in the scale are either forced to change or could not be assessed over a short period.

There are no specific outcomes proposed when comparing methods of reduction. This analysis will focus on the above measures and is exploratory. See Table 3 for a breakdown of which outcomes are to be measured at specific time-points [49].

**Table 3:** Schedule of trial measures

Follow-up	Trial measures
Baseline (wk -2)	Smoking history, demographics, nicotine dependence using Fagerstrom Test for Nicotine Dependence (FTND)[49], urges and withdrawal (MPSS[51]), confidence in quitting, smoking stereotypy[48], cigarette satisfaction[47], future orientation questionnaire, exhaled carbon monoxide (CO), salivary cotinine, Give out diaries.

Pre-quit visit (wk -1)	CO, cotinine, MPSS, confidence, smoking stereotypy, cigarette satisfaction, adverse events. Collect diaries with daily smoking, NRT use, MPSS urge questions. Give out diaries.

Extra pre-quit visit (wk -1a)	**Only applicable if delays quit date by 1 week** CO, MPSS, confidence, smoking stereotypy, cigarette satisfaction, adverse events. Collect diaries with daily smoking, NRT use. Give out diaries.

Extra pre-quit visit (wk -1b)	**Only applicable if delays quit date by 2 weeks** CO, MPSS, confidence, smoking stereotypy, cigarette satisfaction, adverse events. Collect diaries with daily smoking, NRT use. Give out diaries.

Quit day (wk 0)	CO, cotinine, MPSS, confidence, smoking stereotypy, cigarette satisfaction, adverse events. Collect diaries with daily smoking, NRT use, MPSS urge questions. Give out diaries.

One week after quit day (wk +1)	CO, cotinine, MPSS, confidence, cigarette satisfaction if lapsed, adverse events Collect diaries with daily smoking, NRT use, MPSS urge questions

Post-quit visits (wks +2, +3, +4)	Smoking in past week, CO, MPSS, confidence, cigarette satisfaction if lapsed, future orientation questionnaire at week 4, amount NRT used, adverse events

+8 wk visit	Smoking in past 4 weeks, CO, MPSS, cigarette satisfaction if lapsed, confidence, amount NRT used, adverse events

6-month telephone call	Smoking status over past 5 months, use of NRT. Those claiming 7-day abstinence will be invited to a validation visit for exhaled CO and to complete the future orientation questionnaire. Serious adverse events (SAEs)

In addition to the weekly clinic measures, participants will complete a daily diary for the first three weeks of the trial from week -2 to week +1. This will record whether or not the patch is worn, the amount of acute NRT used, the number of cigarettes smoked, and the two urge to smoke questions from the Mood and Physical Symptoms Scale (MPSS). Once a week, the diary will include the mCEQ, with the other measure being taken in clinic.

### Trial Statistics

#### Power calculation

We propose a non-inferiority trial following the Consolidated Standards of Reporting Trials (CONSORT) statement for such trials[29]. With a one-sided alpha of 0.05 we have 80% power to detect inferiority of 9.5% in the quit rate at four weeks if we enrol 343 in each arm, assuming 50% 4-week abstinence. Therefore the trial sample size shall be N = 700. Analyses will be carried out on an intention-to-treat basis, according to the Russell Standard, where participants lost to follow up are assumed to be smokers [20].

Arguably, differences of 5% at 4 weeks would be worthwhile detecting[50], but a trial would need to enrol 2500 participants for this, which would make it impractical and unlikely to be funded. If the reduction produced abstinence rates not worse than 9.5% less at four weeks, this is probably sufficient for stop smoking services to implement the programme.

#### Analysis

The analysis will compare the proportions stopping smoking, calculating confidence intervals and relative risks and confidence intervals. We will calculate differences in withdrawal scores using regression, controlling for baseline differences, as is standard using the MPSS[51]. We will investigate possible mechanisms of action (NRT dose, satisfaction from smoking, withdrawal, self-efficacy) using mediation analysis within a regression framework. Mediation analysis is outlined in more detail by the RIPL research group at the Arizona State University[52].

In comparing methods of reduction, we have no specified hypotheses. We will compare changes in confidence, stereotypy, and urges to smoke calculating differences in mean changes and confidence intervals for the difference. We will compare the proportion abstinent using point prevalence and prolonged abstinence as in the comparison between reduction and abrupt cessation. The primary analysis of abrupt cessation versus reduction will not be adjusted by method of reduction used.

#### Trial schedule

With two fulltime nurses who will also act as trial co-ordinators and liaise with practices, and do follow ups, and the support of a PhD student, we can see the 700 participants in 24 months (bearing in mind that clinical contacts span 10 weeks).

#### Definition of end of trial

End of trial is defined as the final 6 month patient follow-up measuring carbon monoxide level of the last participant undergoing the trial.

### Safety Reporting

#### Assessment of safety

Potential participants' safety will be ensured by screening for eligibility using a structured form completed by the trial nurse. This will record evidence of eligibility and exclusion criteria. In addition, the nurse will take a general medical history to assess for other complicating diseases. Any queries remaining as a result of this process will be resolved by discussion between the trial nurse, chief investigator and the relevant physicians providing routine medical care, usually the participant's general practitioner. Such concerns are unusual but not rare. Typically, they arise from a participant's hazy knowledge or understanding of their past medical history and are usually readily resolved. No blood or further medical testing will be necessary to ensure safety.

NRT has been investigated in several hundred previous clinical trials and is widely prescribed worldwide and subject to safety monitoring, and is replacing a product, nicotine, which the participants are already consuming and will have consumed for many years in cigarettes. Thus, there is every reason to expect that treatment in this trial will be safe. Participants will be warned about the side-effects of NRT and advised not to stop taking the medication without consulting with an NHS professional, preferably the trial team. To this end, all participants will be given a credit card-sized card with the trial team's contact details on that will allow participants to receive advice on medication or to report perceived serious adverse effects and receive advice on medication as required. Participants will record the occurrence of side-effects of medication as specified on the summary of product characteristics for relevant NRT preparations, by completing a checklist. The checklist will be given to the trial nurse and the nurse will enquire about recorded adverse events, so as to determine the severity of any adverse event and ensure that appropriate advice is given for its management (such as rotating the patch site or use of emollients for skin reactions). Minor adverse reactions will be monitored and managed in this way. For each known side effect listed in the summary of product characteristics, the trial nurse will have a definition of clinical severity. For example, a mild skin site reaction to the patch will be defined as burning sensation that does not interfere with normal activities, redness or swelling at the site of application, or mild blistering. Any reaction beyond that will be classified as potentially moderate or severe and will be reported to and discussed with the principal investigator. A decision on stopping therapy will then be made with the participant, attending clinician, principal investigator, and other relevant parties as appropriate. Nicotine has a short half life (2 hours), meaning that the blood concentration will not build up during the course of treatment so that new side-effects are not expected after the first few weeks. In addition, with reactions relating to local use, such as skin discomfort from patches, or bad taste from oral use, either treatment will have been switched or people become accustomed to the side-effects after a short time of using the preparation. At the last meeting (week 8 of the quit attempt), the participant will be advised to phone the contact number to report side-effects that occur after this. The advice given will depend upon the severity of the reported reaction and those with moderate reactions will be invited to an ad hoc consultation.

Participants will also complete a schedule of nicotine overdose symptoms at each visit. On completion of this questionnaire, the schedule will be handed to the nurse and thus any symptoms of overdose will be assessed. In addition, based on her/his enquiry, the nurse will make an assessment of whether the NRT dose is too high or not, and then what action was taken, such as continue with prescribed dose, or direct the participant to use a lower dose, which will be recorded.

The summary of product characteristics for the relevant NRT products contain no warnings about serious adverse reactions except rare allergic reactions, such as angioedema, and cardiac arrhythmias, occurring in less than 1/1000 users. Thus we expect no or very few suspected unexpected serious adverse reactions (SUSARs) in this trial. The long history of use in and outside of trials for NRT means that SUSARs are unlikely. On the reverse of the trial card given the contact number for advice on side-effect management, there will be instructions for the reporting of serious adverse events. Through direct contact from the participant or contact from their attending physician, we expect to become aware of serious adverse events. If any member of the trial team becomes aware, they will inform the PI within 24 hours. The principal investigator will then assess the seriousness, causality, expectedness and severity of the adverse effects. An immediate decision will be made on the interim use of medication for that participant. If an event is judged severe, it will be reported to the trial sponsor, who will report the event to the Research Ethics Committee and Medicines and Healthcare Regulatory Authority. Participants will be asked weekly to report inter-current illnesses and the response recorded. If any of these inter-current illnesses contra-indicates NRT, this will be immediately reported to the principal investigator and a decision made about continued use of the NRT product. The reporting procedures and definitions are presented in Additional file 1.

#### Monitoring and audit

The progress of the trial will be monitored by quarterly review of records. This will ensure that consent is being obtained and the inclusion and exclusion criteria are adhered to. The medication dispensed and the instructions for using it will also be assessed.

Data cleaning will take place by a series of logical checks on the electronic data. (For example, a person cannot be recorded as prolonged abstinent smoker at 6 months if they were not in such a state at 8 weeks). Discrepant records will be checked with the source documents and the database amended if necessary.

The trial will be potentially subject to audit by the appropriate regulatory authorities and therefore participants will be asked to consent to allow their records to be viewed.

### Data management

The trial is being run as part of the portfolio of trials in the Primary Care Clinical Research and Trials Unit (PCCRTU), a National Institute for Health Research (NIHR) recognised trials unit in Primary Care Clinical Sciences at the University of Birmingham. The data management will be run in accord with the standard operating procedures, which are fully compliant with the Data Protection Act and International Conference on Harmonisation (ICH) Good Clinical Practice (GCP). The source documents for the trial will be the case report forms which will be stored in the trials unit in a locked cabinet in a locked office in a locked department. The trial database will be securely held and maintained by the PCCRTU. On completion of the trial and data checking, the case report forms will be transferred to Modern Records, a secure archiving facility at the University of Birmingham, where they will be held for 15 years and then destroyed. The database will be anonymised and a secure compact disc containing the link between identification number and patient identifiable information will be stored in modern records.

### Data protection and confidentiality

Data will be kept in accordance with the Data Protection Act and the trial registered with the Data Protection Act website at the University of Birmingham. The standard operating procedures of the trials unit will be followed, which are designed to protect patient confidentiality. Patient identifiable data will be shared only within the clinical team on a need-to-know basis to provide clinical care and ensure good and appropriate follow up. Patient identifiable data will also be shared with the general practitioner and approved auditors from the Research Ethics Committee, NHS Research and Development, or the Medicines and Healthcare Regulatory Authority will also be able to see patient identifiable information. Otherwise, confidentiality will be maintained and no one outside the trial team will have access to either the case report forms or the database.

### Ethics and Research Governance

The trial will be conducted in compliance with the principles of the Declaration of Helsinki (1996), the principles of ICH-GCP and run in accord with EU Clinical Trials Directive and all of the applicable regulatory requirements. The study protocol and other documentation have been reviewed and approved by the National Research Ethics Committee (08/H0408/213), the Medicines & Healthcare Regulatory Authority, and local NHS Research & Development offices. Any subsequent protocol amendments will be submitted to the Research Ethics Committee for approval, and the other bodies if necessary. We will comply with ICH-GCP Guidelines over the reporting of adverse events, serious adverse events and suspected unexpected serious adverse reactions (SUSARS). In addition we will provide the Research Ethics Committee with progress reports as well as a copy of the Final Study Report.

### Finance

The study will be funded by the British Heart Foundation and service support costs will be claimed via the Comprehensive Clinical Research Network.

### Publication

The trial results will be written up for submission to a peer reviewed journal and the trial is registered with controlled-trials.com. No data relating to individuals will be identified in these publications.

## Competing interests

PA has done consultancy work for Pfizer, McNeil, and Xenova/Celtic. NL has no competing interests. JTI has no competing interests. JI has no competing interests. JB has no competing interests. RW undertakes research and consultancy for companies that develop and manufacture medications for smoking cessation including nicotine replacement therapy. He also has a share of a patent on a novel nicotine delivery device. SM has no competing interests.

## Authors' contributions

NL participated in the design of the study and drafted the manuscript. PA conceived of the study, participated in its design and helped to draft the manuscript. JTI participated in the design of the study and helped to draft the manuscript. JI, JB, RW, and SM all participated in the design of the study. All authors read and approved the final manuscript.

## Supplementary Material

Additional file 1 **Appendix 1**. Adverse Event Reporting. Click here for file
